# The types of tumor infiltrating lymphocytes are valuable for the diagnosis and prognosis of breast cancer

**DOI:** 10.3389/fgene.2022.1019062

**Published:** 2022-10-25

**Authors:** Ying Sun, Chunyan Zhang

**Affiliations:** Radiology Department, Beijing Shijitan Hospital, Capital Medical University, Beijing, China

**Keywords:** breast cancer, tumor infiltrating lymphocytes, diagnostic immune risk score, prognostic immune risk score, pathway enrichment analysis

## Abstract

This study aimed at constructing a diagnostic immune risk score (dIRS) system and a prognostic immune risk score (pIRS) system for diagnose and prognosis of breast cancer (BC). The gene expression data of BC were downloaded from TCGA dataset (training set), and from GSE65194, GSE29044, GSE42568, and GSE20685 (validation sets). Then, the immune cell type proportions in each dataset were assessed using EPIC tool, and the dIRS system was built based on the SVM-RFE and RF-VIMP algorithms. Subsequently, the pIRS system and the nomogram survival model were established separately using penalized and rms packages. Finally, the differential expressed genes (DEGs) between low and high pIRS groups were screened, and submitted for functional analysis. The dIRS system consisted of B cells, CD8 + T cells, endothelial cells, NK cells, and other cells had high accuracy in distinguishing BC patients from the healthy controls (AUROC >0.7). Subsequently, the pIRS system with the five prognosis-associated immune-infiltrating cell was constructed, and Kaplan-Meier analysis demonstrated that the survival rate of low pIRS group was significantly higher than that of high pIRS group (*p* < 0.05). Based on age, pathologic stage and the pIRS values, the nomogram survival model was built. The AUROC value, Specificity value, Sensitivity value and C-index of the nomogram survival model were higher than 0.7000, and had a good predictive ability for BC. Finally, a total of 539 DEGs were identified, and significantly enriched in six pathways. The dIRS system and the pIRS system composed of immune cells might be critical for the diagnosis and prognosis of BC patients.

## Introduction

As a frequent malignant tumor, breast cancer (BC) arises from the epithelial tissues of the breast (Holen et al., 2017). BC patients often suffer from skin changes, axillary lymphadenoma, nipple discharge, breast deformation, and breast lumps (Moodley et al., 2018; Wani et al., 2018). As the tumor with the highest morbidity in women, BC results in the death of over half a million patients annually worldwide (Winters et al., 2017). Metastatic BC cannot be cured through resection operation, and the early detection is an important way to improve the prognosis and reduce the mortality of BC (Medford et al., 2018). Therefore, more researches should be made to diagnose and treat BC patients as early as possible.

Tumor infiltrating lymphocytes (TILs) are lymphocytes isolated from tumor tissues, which mainly include T cells, natural killer (NK) cells, B cells, and macrophages (Denkert et al., 2018). TILs help to comprehensively understand the tumor immune microenvironment and guide the individualized immunotherapy of tumors (Asano et al., 2017). TILs play roles in killing tumor cells, while their killing capability can be inhibited by multiple factors in the tumor microenvironment or by too few TILs (Tomioka et al., 2018). Among the various cell types associated with the development and progression of cancer, the effects of TILs on prognosis have been extensively studied. Previous studies have shown that the assessment of the extent of tumor infiltration of lymphocytes is an important complementary marker for predicting the recurrence and mortality of tumor patients (Pages et al., 2018; Wang et al., 2018). Non-lymphocyte immune cells are also contained in tumors, which are thought to have unique effects on clinical survival in various tumor types and stages (Jang et al., 2017). However, traditional methods (such as immunohistochemistry or flow cytometry) for detecting tumor immune cell infiltration cannot fully evaluate the impacts of different immune cell types or cannot effectively differentiate the closely related cell populations.

Increased amounts of TILs indicate the response of neoadjuvant chemotherapy in BC patients, which can be applied for selecting the patients suitable for neoadjuvant chemotherapy (Issa-Nummer et al., 2014). Stromal TILs have critical prognostic values in early-stage triple-negative breast cancer (TNBC), which can promote the prognosis of stage I TNBC patients without adjuvant chemotherapy (Park JH et al., 2019). In addition, the diagnostic immune risk score (dIRS) system and the prognostic immune risk score (pIRS) system are two novel immune models, and can provide more effective biomarkers for the diagnosis and prognosis of cancer patients. A previous study of Zhou et al. (2019) established a dIRS model based on the immune cells, as well as found that the significant stepwise increase in dIRS values from normal colon to polyp and tumor tissues, and the high area under the receiver-operator characteristic curve (AUC) values not only indicated that the dIRS model could effectively identify colon cancer patients from individuals with colon polyps and healthy controls, but also demonstrated that the immune system was involved in colon cancer development. Furthermore, it was also found that the pIRS model could predict the response to immunotherapy in colon cancer patients (Zhou et al., 2019). Another study also reported that the dIRS and pIRS features could be used as biomarkers for early diagnosis and survival prediction in digestive system cancers (Yang et al., 2019). Nevertheless, the immune risk score system based on immune-infiltrating cell types has not been built for BC patients. In this study, multiple gene expression profiles of BC were downloaded and analyzed comprehensively. Besides, the proportion of immune cells in each dataset was quantitatively evaluated. Based on the constitutive characteristics of the immune cells in the samples, the dIRS system and the pIRS system for BC were established. Our findings might provide more powerful markers for the early diagnosis and accurate prognosis of BC patients.

## Meterials and methods

### Data downloading and data preprocessing

The gene expression data of BC (downloaded on 10 November 2019; platform: Illumina HiSeq (1217 and 2000) samples) in The Cancer Genome Atlas (TCGA) database (https://cancergenome.nih.gov/) were downloaded. Among the 1,217 samples, 1,108 samples were included in this study after corresponding to the clinical information (such as time, overall survival, pathologic M, pathologic N, pathologic T, pathologic stage, histology type, estrogen receptor [ER] status, Human epidermal growth factor receptor 2 [HER2] status, and partial response [RP] status), including 1009 BC samples and 99 normal samples (the training set).

Meanwhile, appropriate datasets were selected from Gene Expression Omnibus (GEO) database (http://www.ncbi.nlm.nih.gov/geo/) according to the following criteria: 1) the datasets were gene expression profiles; 2) the test objects were solid tumor tissue samples of BC patients (not blood, cell lines, etc.); 3) the detection platform was GPL570 or GPL96 (Affymatrix platform); 4) there were control tissues; 5) the datasets were human expression profiles; 6) the total sample size was no less than 100. Finally, three datasets, including GSE65194 (153 BC samples and 11 control samples; platform: GPL570 Affymetrix), GSE29044 (73 BC samples and 36 control samples; platform: GPL570 Affymetrix), and GSE42568 (104 BC samples and 17 control samples; platform: GPL570 Affymetrix) were obtained for the establishment of dIRS. Besides, GSE20685 (including 327 BC samples with survival prognosis information) was downloaded for building the pIRS system.

### Quantitative evaluation of immune cell type proportion

Estimate the Proportion of Immune and Cancer Cells (EPIC) (Racle J et al., 2017) is a tool used for analyzing the infiltration ratio of immune cells (including B cells, CD4 + T cells, cancer-associated fibroblasts [CAFs], CD8 + T cells, macrophages, endothelial cells, and NK cells) according to the expression data. The expression data of each dataset was uploaded to EPIC tool (https://gfellerlab.shinyapps.io/EPIC_1-1/). For each sample, the final output estimate was normalized to 1, and thus could be directly interpreted as a cell fraction for comparison between different immune cell types and datasets.

### Diagnostic analysis

Based on the quantitative evaluation proportions of immune cells in the training set (TCGA), Support Vector Machine (SVM)-Recursive Feature Elimination (RFE) and Random Forest Variable Importance (RF-VIMP) algorithms were used to screen the infiltration types of characteristic immune cells. SVM-RFE (Lu et al., 2016) is a sequence backward selection algorithm based on the maximum interval principle of SVM, which is used to select the required features factors. Using the e1071 version1.7-1 (Liu and Wang, 2015) and caret version 6.0–76, (Fichou D et al., 2016), packages in R, the optimal combination of characteristic immune cells (parameter: cross, 100-fold cross validation) was selected. The results with the highest accuracy in cross validation were selected as the optimal combination of characteristic diagnostic immune cells. RF-VIMP tests the performance of the generated random forest with the data outside the bag (Breiman, 2001; Ishwaran and Lu, 2019; Ishwaran et al., 2021). Using the bootstrap algorithm of the randomForest package version 4.6–14 in R (Ishwaran et al., 2021), the optimal combination of characteristic immune cells was screened. The results of the bootstrap algorithm with the lowest out of bag error rate were used as the optimal combination of characteristic diagnostic immune cells.

Subsequently, the elements included in the optimal combinations screened by SVM-RFE and RF-VIMP algorithms were integrated, and their intersection was obtained to construct the dIRS system of BC. Then, area under the receiver-operator characteristic (ROC) curve (AUROC) (Krupinski, 2017) was used to evaluate the efficiency of the dIRS system in both the training set and the validation sets (GSE65194, GSE29044, and GSE42568). After that, sensitivity (Sen), positive prediction value (PPV), specificity (Spe), and negative prediction value (NPV) were calculated for ROC curves using the pROC package version 1.12.1 (Robin et al., 2011) in R.

### Prognostic analysis

For the BC samples of the TCGA training set, the Cox-Proportional Hazards (Cox-PH) model in the LASSO algorithm of the R penalized package (version 0.9–50, http://bioconductor.org/packages/penalized/) was used to screen the optimal prognosis-associated immune-infiltrating cell types (parameter: 1,000 fold cross-validation likelihood). Based on the immune-infiltrating cell types and prognostic coefficients, the pIRS system was built as follows:

$$pIRS=\sum \beta_{immune}\times Immune\;cell\;types\;value$$

Where β represented prognostic coefficient of each immune-infiltrating cell type proportion; and “Immune cell types value” represented the proportion values for each immune-infiltrating cell type.

Following that, effectiveness evaluation for the pIRS system was performed in the training set and the validation set GSE20685. Firstly, the pIRS value of each sample in the training set was calculated, and then combined with the survival prognostic information (time and status) of each sample, cutoff Finder (Budczies et al., 2012) was used to obtain the pIRS point with the most significant log-rank test. The log-rank test is the most commonly-used statistical test for comparing the survival distributions of two or more groups. According to this pIRS point, the samples were classified into low (pIRS value <0) and high (pIRS ≥0) pIRS groups (Budczies et al., 2012). Then, the Kaplan-Meier (KM) method in survival package vrsion2.41-1 (Noura and Read, 2018) was used to assess the correlation between the low/high pIRS groups and the actual survival prognosis. At the same time, the proportion of the target immune-infiltrating cell types and pIRS values were also calculated in the samples of the validation set GSE20685. Based on the obtained pIRS value, low (pIRS value <0) and high (pIRS ≥0) pIRS groups were classified, and KM method was also applied for evaluating the correlation between the risk grouping and the actual prognosis information in the validation set GSE20685.

### Establishment of nomogram survival models

Combined with the univariate and multivariate Cox regression analyses in the survival package (Noura and Read, 2018), the independent clinical prognostic factors (age, pathologic M, pathologic N, pathologic T, pathologic stage, histology type, ER status, HER2 status, PR status, vital status, and overall survival time) in the training set were selected. The clinical factors with log-rank *p*-value < 0.05 were considered as the significant difference.

To further reveal the correlation between the independent clinical prognostic factors and the pIRS model, rms package version 5.1–2 (Eng et al., 2015)in R was utilized to build 3-year and 5-year nomogram survival models based on the independent clinical prognostic factors and the risk information discriminated by the pIRS model.

### Differential analysis and pathway enrichment analysis

In the training set, the BC samples were divided into low and high pIRS groups, and then the differential expressed genes (DEGs) between the low and high pIRS groups were screened using limma package version 3.34.7 (Ritchie et al., 2015) in R based on the thresholds of |log_2_ fold change (FC)| > 0.5 and false discovery rate (FDR) < 0.05. FC, fold change of expression value, is used to describe the degree of change from an initial value to a final value, and is often used to measure the levels of gene expression. FDR means adjusted *p* value. After that, all the identified DEGs were subjected for Kyoto Encyclopedia of Genes and Genomes enrichment analysis using Gene Set Enrichment Analysis (GSEA) (Suárez-Fariñas M et al., 2010) with the criterion of FDR <0.05.

## Results

### The proportion of immune cells in each dataset

**FIGURE 1:**
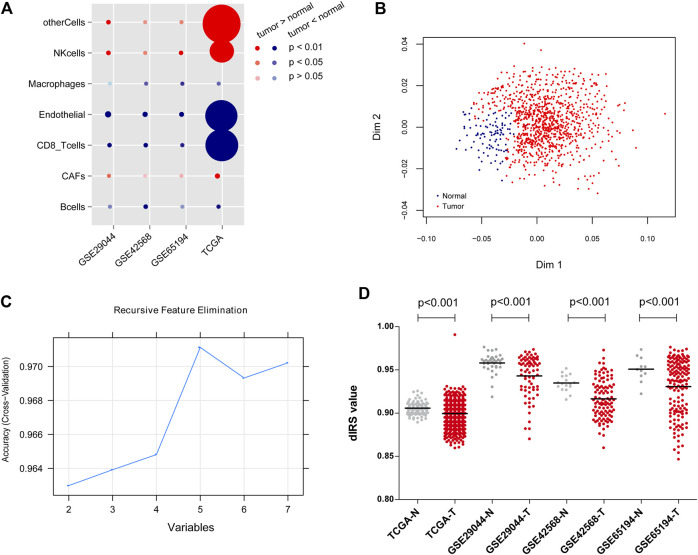
The classifications of immune-infiltrating cells in the datasets, and the construction of diagnostic immune risk score (dIRS) system. **(A)** The bubble diagram showing the proportion differences of the immune-infiltrating cells between the tumor samples and normal samples in each dataset. The red circles indicate that tumor samples have higher proportions of immune-infiltrating cell types than normal samples. The blue circles indicate that tumor samples have lower proportions of immune-infiltrating cell types than normal samples. The darker the red or blue, the more significant the difference. The larger the circle, the higher the proportion of the cell type. **(B)** The scatter diagram for sample classification based on Random Forest Variable Importance algorithm. Blue and red dots separately represent normal samples and tumor samples. **(C)** The line chart for variables based on Support Vector Machine-Recursive Feature Elimination algorithm. **(D)** The distribution of dIRS values in the training set and the validation sets. Grey and red dots represent normal samples and tumor samples, respectively. TCGA, The Cancer Genome Atlas.

Based on EPIC tool, the proportion of immune cells in each dataset was quantitatively assessed, and then the classifications of immune-infiltrating cells in the datasets were compared. As shown in Figure 1A, the proportions of CFAs, NK cells and other cells were consistently higher in the BC tissues than those in the normal tissues; whereas the proportions of B cells, CD8^+^ T cells, endothelial cells, and macrophages were significantly lower in the BC tissues compared with the normal tissues (*p* < 0.05).

### Immune cells for the establishment of the dIRS system to diagnose BC

Based on the SVM-RFE and RF-VIMP algorithms, five immune-infiltrating cell types (B cells, CD8^+^ T cells、endothelial cells, NK cells, and other cells) and seven immune-infiltrating cell types (B cells, CAFs, CD8+T cells, endothelial cells, macrophages, NK cells, and other cells) were screened, respectively. The RF-VIMP algorithm analysis (Figure 1B) and SVM-RFE algorithm analysis (Figure 1C) revealed five overlapping cell types between the two methods, including B cells, CD8^+^ T cells, endothelial cells, NK cells, and other cells.

**FIGURE 2:**
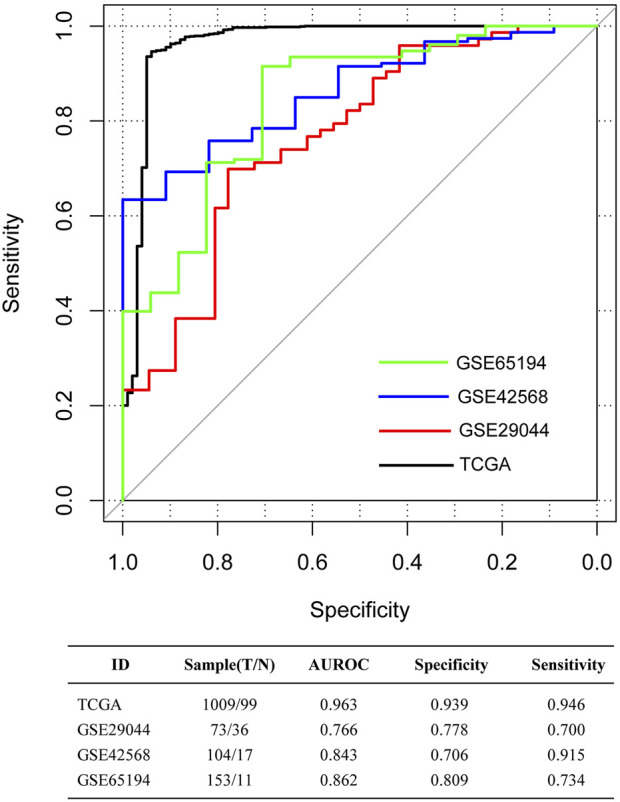
The receiver-operator characteristic (ROC) curves for The Cancer Genome Atlas (TCGA) dataset, and the validation sets GSE65194, GSE29044, and GSE42568. Black, red, blue, and green curves separately represent the TCGA dataset, GSE29044, GSE42568, and GSE65194. The data at the bottom of the figure represents the parameters of ROC curves. AUROC, area under the receiver-operator characteristic curve.

The aforementioned five immune-infiltrating cell types were used for the establishment of the dIRS system to diagnose BC. In this model, the proportions of the selected immune cells were accessed as continuous variables. Figure 1D showed that the dIRS value was significantly decreased in the BC tissues compared with the normal tissues in both the training set and the validation sets (*p* < 0.001). In addition, we evaluated the efficiency of the dIRS system, and the results of ROC curves for the training set (AUROC = 0.963, Spe = 0.939, Sen = 0.946) and the validation sets (GSE29044: AUROC = 0.766, Spe = 0.778, Sen = 0.700; GSE42568: AUROC = 0.843, Spe = 0.706, Sen = 0.915; GSE65194: AUROC = 0.862, Spe = 0.809, Sen = 0.734) showed that the dIRS system had high accuracy in distinguishing BC patients from the healthy controls (AUROC >0.7, Figure 2).

### Immune cells of the construction of the pIRS system to predict BC prognosis

**FIGURE 3:**
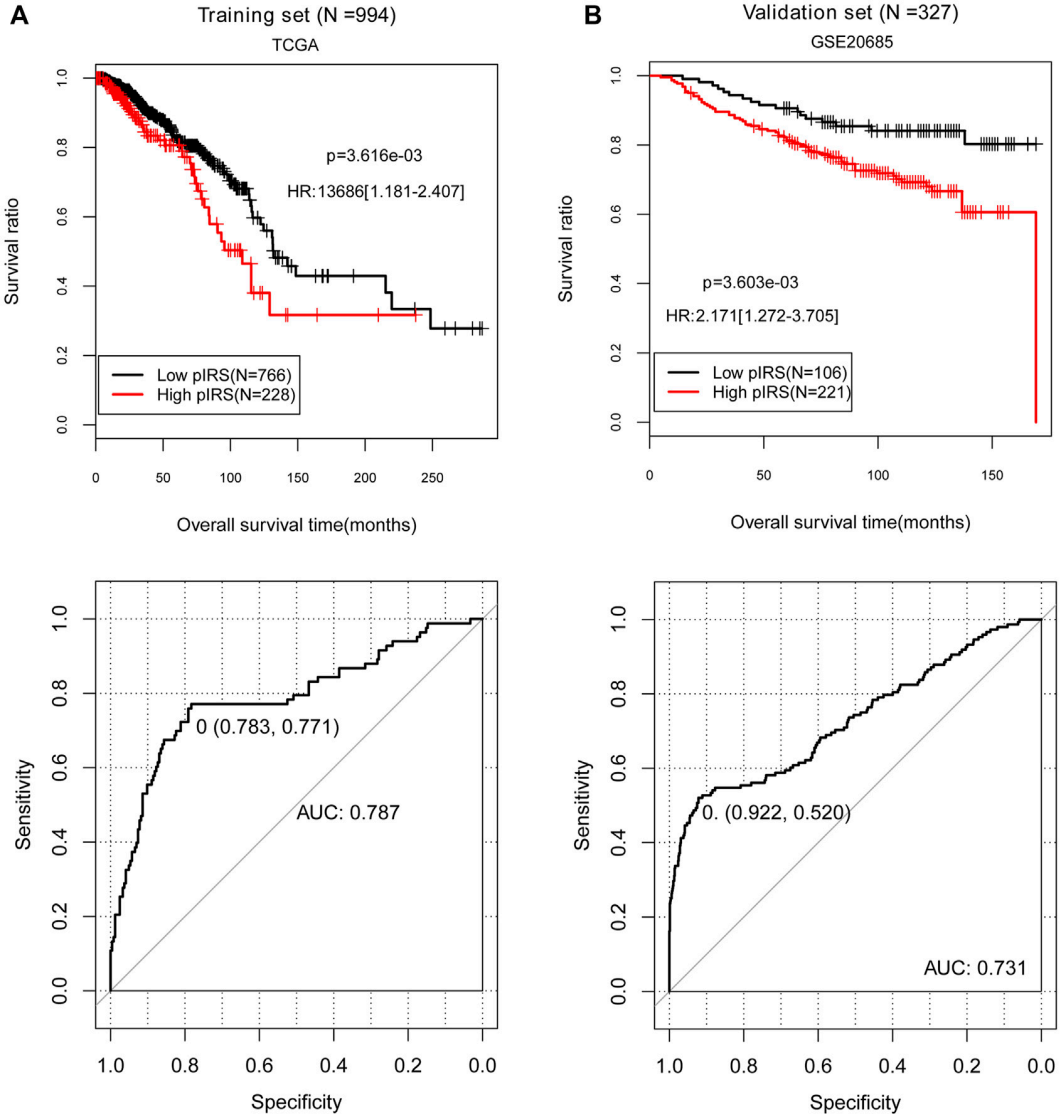
The Kaplan-Meier (KM) curves and receiver-operator characteristic (ROC) curves showing the correlation of low/high prognostic immune risk score (pIRS) groups and the actual survival prognosis. **(A)** The KM curves (above) and ROC curve (below) for the training set. **(B)** The KM curves (above) and ROC curve (below) for the validation set GSE20685. In KM curves, black and red curves separately represent the samples in low and high pIRS groups. In ROC curves, the points marked in the figure indicate the corresponding specificity and sensitivity values when pIRS value is the cutoff point (0). TCGA, The Cancer Genome Atlas; AUC, area under the receiver-operator characteristic curve.

In the 994 BC tissue samples with survival and prognosis information in the training set, five prognosis-associated immune-infiltrating cell types were identified using the LASSO algorithm, containing B cells, endothelial cells, macrophages, NK cells, and other cells (Table 1). Then, the pIRS system was constructed based on these five prognosis-related immune-infiltrating cell types. According to the cut-off value obtained in the entire training set (0), we divided the samples into low and high pIRS groups, and there were 766 samples and 228 samples respectively in the low and high pIRS groups (Figure 3A). Similarly, the samples in the validation set (GSE20685) were also divided into low and high pIRS groups with 106 and 221 samples, respectively (Figure 3B). Moreover, the KM curves in the training set and the validation set both suggested that the BC patients with the low pIRS had better clinical prognosis compared to the high pIRS (*p* < 0.05, Figures 3A,B). The AUROC values in the training set and the validation set were respectively 0.787 and 0.731, which indicated the built pIRS system with the five prognosis-associated immune-infiltrating cell types was good, and was well verified in validation dataset due to the consistency with that in the training set (Figures 3A,B).

**TABLE 1:** The prognosis-associated immunoinfiltrating cell types identified by LASSO algorithm.

Symbol	Multi-variate cox regression analysis			
	HR	95% CI	p-value	LASSO coefficient
B cells	0.468	0.319–0.684	8.89E-05	−0.5092
Endothelial	1.203	1.077–1.649	2.52E-02	0.0908
Macrophages	2.188	1.447–3.052	2.73E-03	0.0912
NK cells	1.021	1.005–1.219	8.16E-03	0.0252
Other Cells	0.827	0.679–0.911	6.64E-03	−0.0216

Note: HR, hazard ratio; CI, confidence interval; NK, natural killer.

### Identification of independent clinical factors related to prognosis and assessment of a nomogram survival model

**FIGURE 4:**
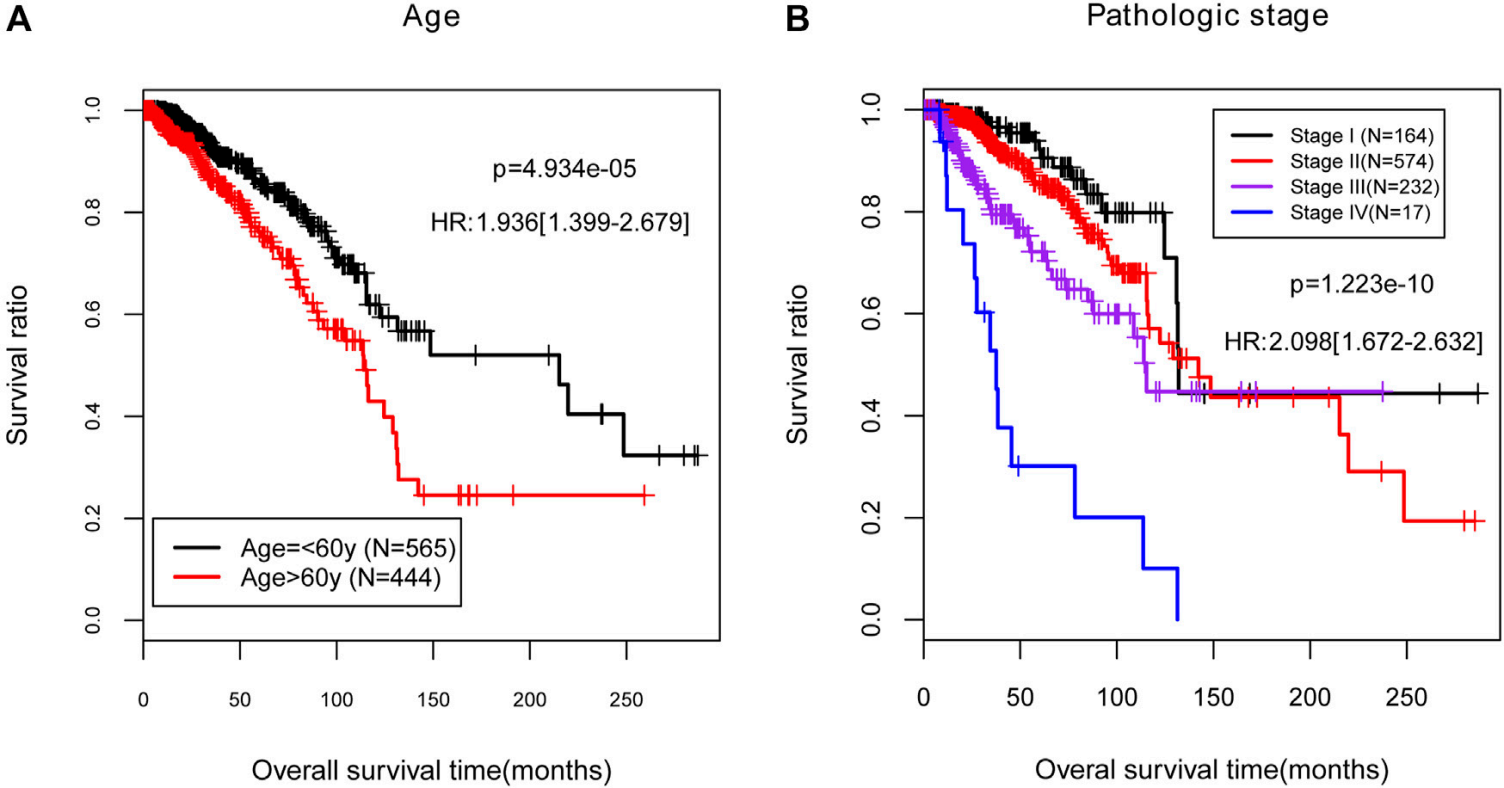
The Kaplan-Meier (KM) curves for the independent clinical prognostic factors in the training set. **(A)** The KM curves for age (black and red curves separately represent the samples aged no more than 60). **(B)** The KM curves for pathologic stage. Black, red, purple, and blue curves represent the samples in stage I, stage II, stage III, and stage IV, respectively. HR, hazard ratio.

To better clarify the correlation between the independent clinical factors and recurrence prognosis, we developed a nomogram survival model. According to the results of Cox regression analyses, age and pathologic stage were selected as the significantly independent clinical prognostic factors in the training set (*p* < 0.05, Table 2). Then we studied on the relationship between age or pathologic stage and recurrence prognosis, the result showed that the BC patients aged below 60 years old and with lower pathologic stages (Stage I, Stage II, and Stage III) had higher survival ratio (*p* < 0.05), which was consistent with the actual situation (Figures 4A,B).

**TABLE 2:** The screening results of the independent clinical prognostic factors in the training set.

Clinical characteristics	TCGA (N = 1,009)	HR	Uni-variables cox		HR	Multi-variables cox	
			95%CI	P		95%CI	P
Age (years, mean ± SD)	58.32 ± 13.20	1.031	1.018–1.043	1.66E-06	1.033	1.018–1.047	5.34E-06
Pathologic M (M0/M1/-)	875/19/115	4.111	2.385–7.084	3.59E-08	1.294	0.618–2.709	4.94E-01
Pathologic N (N0/N1/N2/N3-)	471/335/114/71/18	1.602	1.345–1.908	7.45E-08	1.184	0.890–1.576	2.47E-01
Pathologic T (T1/T2/T3/T4/-)	255/591/124/35/4	1.439	1.178–1.757	3.45E-04	0.964	0.714–1.302	8.11E-01
Pathologic stage (I/II/III/IV/-)	164/574/232/17/22	2.098	1.672–2.632	1.22E-10	1.744	1.065–2.858	2.72E-02
Histology type (Basal/Her 2/LumA/Lum B/Normal/-)	139/67/420/191/24/168	1.129	0.955–1.336	1.56E-01	—	—	—
ER Status (Positive/Negative/-)	596/178/235	0.995	0.644–1.537	9.81E-01	—	—	—
HER2 Status (Positive/Negative/-)	114/647/248	1.030	0.585–1.811	9.20E-01	—	—	—
PR Status (Positive/Negative/-)	518/253/238	0.926	0.629–1.362	6.96E-01	—	—	—
Vital status (Dead/Alive/-)	148/846/15	—	—	—	—	—	—
Overall survival time (months, mean ± SD)	42.42 ± 40.54	—	—	—	—	—	—

Note: TCGA, the cancer genome atlas; HR, hazard ratio; CI, confidence interval; ER, estrogen receptor; HER2, human epidermal growth factor receptor 2; PR, progesterone receptor; SD, standard deviation.

**FIGURE 5:**
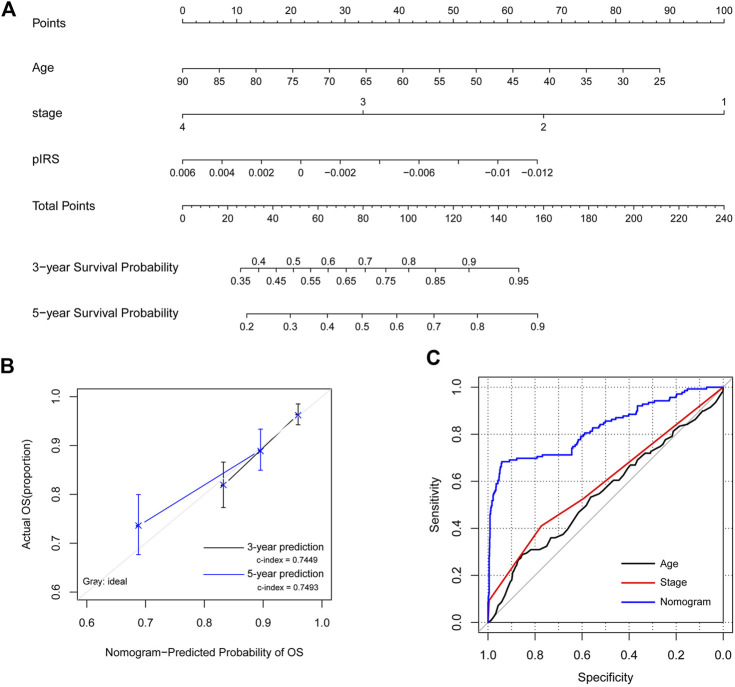
The construction and evaluation of the nomogram survival model. **(A)** The nomogram survival model based on age, pathologic stage, and the prognostic immune risk score (pIRS) values. **(B)** The diagram showing the consistency of the predicted and the actual survival probabilities. **(C)** Comparison of the predictive abilities of the age-based model (black), the stage-based model (red), and the nomogram survival model (blue). OS, overall survival.

To provide a quantitative method to predict the probability of recurrence, a nomogram survival model with the 3-year and 5-year survival probability based on age, pathologic stage and the pIRS model was built (Figure 5A). The 3-year or 5-year survival probability predicted by the nomogram survival model were compared with the actual 3-year or 5-year survival ratio. It was found that the c-indexes for the 3-year prediction and 5-year prediction were 0.7449 and 0.7493, respectively, which were higher than 0.7000, and indicated that the nomogram survival model was reliable (Figure 5B). Besides, the AUROC value, Spe value, Sen value and C-index of the nomogram survival model were 0.826, 0.941, 0.683 and 0.749, respectively, which were higher than those of the age-based model, and pathologic stage-based model (Figure 5C; Table 3). These results implied that the nomogram survival model had a better predictive ability than age-based and pathologic stage-based models.

**TABLE 3:** The parameters for the age-based model, the stage-based model, and the nomogram survival model.

ID	AUROC	Specificity	Sensitivity	C-index
Age	0.549	0.655	0.582	0.641
Stage	0.594	0.673	0.541	0.680
Nomogram	0.826	0.941	0.683	0.749

Note: AUROC, area under the receiver-operator characteristic curve.

### Identification of DEGs between low and high pIRS groups and functional analysis

Based on the criteria of |log_2_ FC| > 0.5 and FDR <0.05, a total of 539 DEGs were identified between the low and high pIRS groups in the training set, including 365 downregulated genes and 174 upregulated genes. After that, these identified DEGs were sent for KEGG pathway enrichment analysis, and with the threshold of FDR <0.05, six significant KEGG pathways were significantly enriched, including cytokine-cytokine receptor interaction (FDR = 1.250E-03), chemokine signaling pathway (FDR = 1.536E-03), B-cell receptor signaling pathway (FDR = 2.481E-02), T-cell receptor signaling pathway (FDR = 1.828E-02), Toll-like receptor signaling pathway (FDR = 1.903E-02), and antigen processing and presentation (FDR = 1.623E-02) (Table 4).

**TABLE 4:** The six significant pathways involving the differential expressed genes.

Name	Size	ES	NES	*p*-value	FDR
KEGG_CYTOKINE_CYTOKINE_RECEPTOR_INTERACTION	23	−0.5505	−2.4319	0	1.250E-03
KEGG_CHEMOKINE_SIGNALING_PATHWAY	16	−0.5553	−2.1649	0	1.536E-03
KEGG_B_CELL_RECEPTOR_SIGNALING_PATHWAY	2	−0.5660	−1.5536	6.221E-03	2.481E-02
KEGG_T_CELL_RECEPTOR_SIGNALING_PATHWAY	4	−0.5795	−1.7560	1.192E-03	1.828E-02
KEGG_TOLL_LIKE_RECEPTOR_SIGNALING_PATHWAY	4	−0.6483	−1.7249	1.553E-03	1.903E-02
KEGG_ANTIGEN_PROCESSING_AND_PRESENTATION	14	−0.4876	−1.6439	3.492E-03	1.623E-02

Note: KEGG, kyoto encyclopedia of genes and genomes; ES, enrichment score; NES, normalized enrichment score; FDR, false discovery rate.

## Discussion

In this study, five immune-infiltrating cells were chosen to construct the dIRS system, and it was found the five immune-infiltrating cell types-consisted dIRS system could distinguish BC patients from normal controls. Then, five prognosis-related mmune-infiltrating cells were identified to build the pIRS system, and the results showed that the pIRS system was good to predict the BC prognosis. In addition, a nomogram survival model was built based on the significantly independent clinical prognostic factors (age and pathologic stage) and pIRS, which could have a better predictive ability for BC. Finally, we identified 539 DEGs between the low and high pIRS groups, which were significantly enriched in six KEGG pathways.

Based on five infiltration types (B cells, CD8 + T cells, endothelial cells, NK cells, and other cells), the dIRS system of BC was constructed in our study, and it was found that the dIRS value was significantly decreased in the BC tissues compared with the normal tissues, and the AUROC values for the training set and validation sets were all above 0.7, which indicated that the dIRS system had high accuracy for BC diagnosis. T follicular helper cell activation of B cells induced by immune checkpoint inhibitors contributes to the anti-tumor response in BC models, and thus T follicular helper cells and B cells are involved in the immunotherapy of BC (Hollern DP et al., 2019). Tumor-infiltrating B-cells (TIL-B) has influences on the improved clinical prognosis of BC patients, which can generate sustained humoral immune responses and effective anti-tumor immunity in the tumor (Garaud S et al., 2019). Through promoting the proliferation and activity of CD8^+^ T cells and making tumor cells sensitive to T-cell recognition, class I histone deacetylase (HDAC) inhibitors damage BC cell growth (McCaw TR et al., 2019). Both CD4^+^ and CD8^+^ T cells are correlated with immune responses, while they have different effects on the disease progression and clinical outcomes of BC patients (Huang et al., 2015). Endothelial cell has interactions with the tumor microenvironment, and its proliferation, invasion, and migration are inhibited by miR-7 expression in BC patients (Cui et al., 2017). The combination of epidermal growth factor receptor (EGFR)-chimeric antigen receptor (CAR) NK-92 cells with oncolytic herpes simplex virus (oHSV) can lead to a higher mortality of MDA-MB-231 BC cells and better outcomes of BC mice, indicating that oHSV-1 therapy combined with EGFR-CAR NK-92 cells is promising in treating the brain metastases of BC (Frings et al., 2011; Chen et al., 2016). Combined with our results, it can be inferred that the dIRS system based on the five immune-infiltrating cell types might be valuable for the diagnosis of BC patients.

The present study also screened five prognosis-associated immune-infiltrating cell types (B cells, endothelial cells, macrophages, NK cells, and other cells) to establish the pIRS system, and the AUROC values of the pIRS system for different datasets were both above 0.7, which manifested that the built pIRS system was good. Tumor-associated macrophages (TAMs) in BC microenvironment function as tumor-promoting cells, which contributes to tumor progression and can induce treatment-resistance in BC models (Cassetta and Pollard, 2017; Qiu SQ et al., 2018). High density of TAMs is significantly related to the malignant phenotype and negative hormone receptor status in BC, and TAMs infiltration can be considered as a prognostic factor in patients with the tumor (Zhao et al., 2017). Additionally, a nomogram survival model was also established using age, pathologic stage and the pIRS system, and ROC curves showed that the nomogram survival model had a better predictive ability. Therefore, we can speculate that the pIRS system based on the five prognosis-associated cell types might be better and effective in predicting the prognosis of BC patients.

Finally, 539 DEGs between the low and high pIRS groups were identified, and were significantly enriched in six significant pathways, including chemokine signaling pathway, B-cell receptor signaling pathway, T-cell receptor signaling pathway, and Toll-like receptor signaling pathway. Overexpressed C-C motif chemokine receptor 2 (*CCR2*) promotes the progression of early-stage BC *via* stromal-dependent C-C motif chemokine ligand 2 (*CCL2*) expression, therefore, chemokine signaling can affect the therapy and outcomes of BC patients (Brummer et al., 2018). B-cell receptor plays important roles in the development and maturation of normal B-cells, and B-cell receptor signaling is involved in the tumorigenesis of various B-cell malignancies (Niemann and Wiestner, 2013). T-cell receptor pathway may be correlated with the pathogenesis of extranodal NK/T-cell lymphoma, and the inducible T cell kinase (*ITK*) involved in T-cell receptor pathway may serve as a candidate target for treating the lymphoma patients expressing *ITK* (Tomoko et al., 2018). The toll-like receptor signaling pathway has correlations with the risk, progression, and survival of BC patients, which provides novel ideas for improving the therapeutic strategies of the disease (Kidd et al., 2013). Thus, the identified DEGs enriched pathways of chemokine signaling pathway, B-cell receptor signaling pathway, T-cell receptor signaling pathway, and Toll-like receptor signaling pathway might be correlated with the progression and prognosis of BC patients. However, the specific roles of these pathways in BC warrant to be further investigated.

Although tumor stage and molecular markers had been applied for the diagnosis and treatment of BC, this study constructed the dIRS system, pIRS system, and nomogram survival model to improve the predictive accuracy of BC. However, our findings need to be further validated by more *in vitro* and *in vivo* experiments in the future. Additionally, the application of the dIRS and pIRS systems to clinical detection methods will be another important task for our future work.

## Conclusion

In conclusion, our study reveals the roles of immune cells in the diagnosis and prognosis of BC. With the rapid development of high-throughput technology, we are confident that our proposed dIRS system and the pIRS system based on the immune-infiltrating cells may have great potential in the diagnosis, treatment evaluation, and prognosis of BC. These findings may provide much-needed comprehensive clinical information to improve the personalized management of BC patients.

## Data Availability

The datasets presented in this study can be found in online repositories. The names of the repository/repositories and accession number(s) can be found in the article/Supplementary Material.

## Abbreviations

BC, Breast cancer; dIRS, diagnostic immune risk score; DEGs, differential expressed genes; TILs, Tumor infiltrating lymphocytes; NK, natural killer; TNBC, triple-negative breast cancer; pIRS, prognostic immune risk score; TCGA, The Cancer Genome Atlas; EPIC, Estimate the Proportion of Immune and Cancer Cells; CAFs, cancer-associated fibroblasts SVM, Support Vector Machine; RFE, Recursive Feature Elimination; ROC, receiver-operator characteristic; Sen, sensitivity; PPV, positive prediction value; Spe, specificity; NPV, negative prediction value; Cox-PH, Cox-Proportional Hazards; TIL-B, Tumor-infiltrating B-cells; HDAC, histone deacetylase; EGFR, epidermal growth factor receptor; CAR, chimeric antigen receptor; oHSV, oncolytic herpes simplex virus; TAMs, Tumor-associated macrophages; CCR2, C-C motif chemokine receptor 2; CCL2, C-C motif chemokine ligand 2; ITK, inducible T cell kinase.
